# Grief and Bereavement in the Latino/a Community: A Literature Synthesis and Directions for Future Research

**DOI:** 10.1089/heq.2022.0031

**Published:** 2022-09-14

**Authors:** Francesca Falzarano, Hillary Winoker, Rebecca V. Burke, Jose A. Mendoza, Francisco Munoz, Ana Tergas, Paul K. Maciejewski, Holly G. Prigerson

**Affiliations:** ^1^ Center for Research on End-of-Life Care, Division of Geriatrics & Palliative Medicine, Weill Cornell Medicine, New York, New York, USA.; ^2^ University of Texas Medical Branch, Galveston, Texas, USA.; ^3^ City of Hope, New York, New York, USA.

**Keywords:** bereavement, grief, Hispanic, Latino, mental health

## Abstract

**Introduction:**

Bereavement and grief are social phenomena influenced by a multitude of cultural factors. Prior studies of bereavement adjustment have primarily focused on bereaved survivors who identify racially as white; knowledge of the experience of grief and bereavement among racial/ethnic and other minority groups, particularly among Latino/a groups, in the United States is limited.

**Objective:**

The purpose of this review is to synthesize the literature documenting the bereavement experiences of the Latino/a community, evaluate the strength of the current evidence, and provide recommendations to guide future research.

**Method:**

A narrative review of research on grief and bereavement in the Latino/a community published between 1990 and 2021. Two authors used a thematic, deductive approach to categorize emergent prevalent themes from the literature and used The Grading of Recommendations Assessment, Development, and Evaluation (GRADE) and The Oxford Center for Evidence-Based Medicine—Evidence Quality Rating Scale (OCEBM) approaches to evaluate the strength of the qualitative and quantitative reports reviewed.

**Results:**

Searches revealed 26 reports that were categorized into six themes: cultural values, mourning rituals, immigration, spirituality, disparities related to the COVID-19 pandemic, and the effects of COVID-19 on Latino/a communities. Our evaluation concludes that the evidence in this area is weak, with limited methodologically rigorous research examining the influence of culture on bereavement among Latino/a groups.

**Conclusion:**

Research is needed to identify Latino/a groups' mental health, cultural, social, and family needs and how fulfillment of mourning rituals and other cultural factors may promote or impede bereavement adjustment. Investigation into factors that may protect bereaved survivors against adverse mental health outcomes is also needed. A better understanding of Latino/a grief and bereavement is a step toward the development of culturally competent interventions designed to promote the mental health and psychosocial adjustment of Latino/a mourners.

## Introduction

Bereavement is, by definition, a social experience because it is the loss of a significant other.^1^ Grief is the emotional response to this social loss, manifesting itself with feelings of yearning and missing the deceased person.^1,2^ Thus, both bereavement and grief are inherently social phenomena that are strongly influenced by social, cultural, historical, and political forces.^2^ Cultural factors contribute to individuals' perceptions of and meanings associated with illness, suffering, dying, and death. The communal aspects of culture, including social norms, rituals, roles, and interpersonal relationships, influence the meaning attributed to and the experience of the mourning process—all of which vastly differ across cultures, religions, and racial/ethnic groups.^2,3^

### Grief from a cultural lens

Although culture can exert distinctive influences on the expression of grief, prior research has also identified crosscultural commonalities in bereavement practices, including the use of rituals and mourning practices to memorialize the deceased. Across cultural groups, the performance of postloss rituals is thought to promote bereavement adjustment, aiding the bereft individual in re-establishing a sense of normalcy following loss.^2,4^ However, existing “culturally sanctioned” models of grief may be problematic as bereavement is context dependent; that is, grief reactions are influenced by the multilevel systems in which individuals are embedded.^4,5^ Thus, what may be viewed as maladaptive bereavement adjustment in one cultural group may be considered normative in another,^6^ which can further perpetuate the likelihood of feeling stigmatized and/or experiencing disenfranchised grief.^5^

Current knowledge about grief and bereavement among racial/ethnic minority groups, particularly among Latino/a Americans (hereafter, referred to as “Latino/a”) in the United States is quite limited. At the time of this writing, to our knowledge, only one systematic review has examined Latino widows in the United States^7^ and identified major methodological weaknesses in studies targeting the bereavement experience in the Latino/a community. More rigorous, methodologically sound research is needed to gain a better understanding of how bereavement is experienced and understood among racial/ethnic minority groups, specifically among Latino/a bereaved individuals. Extending the work of Garcini et al,^7^ we, in this study, present a narrative synthesis of the existing literature documenting the bereavement experiences of the Latino/a community of various types of loss, followed by recommendations for future research.

Research on grief and bereavement has primarily focused on white populations, limiting its generalizability and applicability to racial/ethnic minority groups. The lack of attention to bereavement among Latino/a persons is a major gap given that persons of Hispanic/Latino/a descent are the largest minority group in the United States.^8,9^

Although we will, in this study, consider Latino/a individuals as a group, it is important to recognize that Latino/as are not a monolithic group of people and instead represent a wide variety of cultures and countries of origin (i.e., Mexico, Puerto Rico, Dominican Republic, Cuba, French-speaking Caribbean nations, and Central and South America) with unique customs and beliefs related to grief and loss. Furthermore, although LatinX is widely used to promote gender inclusivity, the term itself is controversial, with few people of Latino/a descent using the term LatinX.^10^ Thus, we use the term Latino/a, in this study. There remains a critical need to begin to understand how grief is experienced by members of this large diverse group of Latino/a individuals.

## Methods

**FIG. 1.**
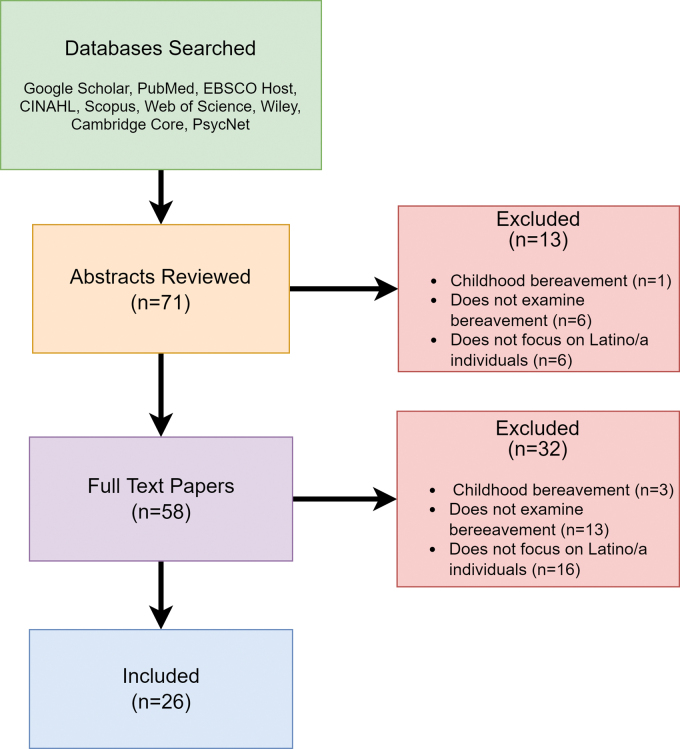
Flowchart of review process.

This narrative review synthesizes literature examining bereavement in the Latino/a community. The review was conducted using the following search terms: “Latino/a bereavement,” “Latino/a grief,” “Hispanic bereavement,” and “Hispanic grief” in PubMed, EBSCO Host, CINAHL, Scopus, Web of Science, Wiley, Cambridge Core, PsycNet, and Google Scholar. We began conducting searches in April 2021 and concluded in October 2021 (articles were last consulted on October 1, 2021). Articles published before 1990 were excluded from the review. In general, liberal criteria were applied for study inclusion (e.g., study design), but only articles focused on grief and bereavement specific to adults in Latino/a communities were included. All abstracts and full-text articles were evaluated by two authors to determine relevance for inclusion in the review. Abstracts and articles were excluded if they (1) focused on bereavement in childhood/adolescent (under the age of 18) Latino/a individuals (*n*=4); (2) did not specifically examine bereavement (*n*=19); (3) and/or did not explicitly focus on the bereavement experience of Latino/a individuals (*n*=22) (Fig. 1).

A deductive, thematic approach was taken to form the structure from which the findings were synthesized.^11,12^ Two members of the research team independently reviewed and synthesized key points from full-text articles (*n*=26), which were used to derive emergent themes in the literature. The reviewers first convened to evaluate and achieve consensus on derived themes, which were used to categorize and synthesize the articles, clustering themes. The reviewers then independently evaluated the strength of the evidence for each article meeting inclusion criteria and convened to discuss ratings and resolve discrepancies to arrive at a final synthesis and evaluation of the strength of the evidence for each article.

For qualitative studies, we rated the strength of the evidence using the Grading of Recommendations Assessment, Development, and Evaluation (GRADE) rating system.^13–15^ The GRADE system evaluates evidence reported in the literature based on four levels (high, moderate, low, and very low) according to the consistency of the findings across studies. For example, GRADE assessments assign the highest grades based on study quality and limitations.^16^ Each source is assigned a checklist quality score based on its strengths and weaknesses (i.e., study design, methodology, statement of findings) using the Critical Appraisal Skills Quality Programme (CASP) Checklist.^17^ Sources are provided a score of “++” if most/all criteria were fulfilled, “+” if some criteria were fulfilled, or “−” if little-to-no criteria are fulfilled).

For quantitative studies, we used the Oxford Center for Evidence-Based Medicine—Evidence Quality Rating Scale (OCEBM).^18^ This system rates the quality of data from 1 to 5 depending on the methods used to collect the data as well as the overall study design. These evaluations were only used to assess methodology and were not used to inform our synthesis. Table 1 presents the OCEBM criteria used to evaluate the empirical literature.

**Table 1.** Modified from the Oxford Center for Evidence-Based Medicine for Ratings of Individual Studies^15^

Quality rating scheme for studies and other evidence	
1	Properly powered and conducted randomized clinical trial; systematic review with meta-analysis
2	Well-designed controlled trial without randomization; prospective comparative cohort trial
3	Case–control studies; retrospective cohort study
4	Case series with or without intervention; cross-sectional study
5	Opinion of respected authorities; case reports

## Results

Table 2 presents an overview of the literature with key demographic characteristics (when available) for each study included in our review. Articles included in the synthesis comprised narrative reviews (*n*=5), qualitative (*n*=11) and quantitative (*n*=6) studies, book chapters (*n*=1), one systematic review, as well as published commentaries (*n*=2). Table 3 presents our evaluation of the strength of the literature.

**Table 2.** Characteristics of Studies for Inclusion in Narrative Synthesis

Search engine	Authors	Sample size	Age	Gender	Key findings	Latino subgroup	Years in United States
Grief from a Cultural Lens							
PubMed, Google Scholar, EBSCOHost	Hardy-Bougere (2008)	NR	NR	NR	Mourning rituals are essential to bereavement adjustment.	Mexico, Cuba, DR, Nicaragua, Columbia, El Salvador, Guatemala, Chile, Brazil, Argentina, Peru	NR
Google Scholar	Clements et al (2003)	NR	NR	NR	Significance of mourning rituals in Latino/a community.	NR	NR
EBSCOHost, Google Scholar, Scopus, CINAHL	Rosenblatt (2017)	NR	NR	NR	There are strong cultural differences in grief processes.	NR	NR
Latino Grief Experience							
PubMed, Google Scholar, Web of Science, CINAHL, Scopus	Brooten et al (2016)	*N*=63	*M*=35.1, SD=9.0	70% female	Postdeath decision making for bereaved parents is especially distressing for immigrant parents with language barriers.	Mexico, Cuba, Haiti, Puerto Rico, Colombia, Ecuador, Nicaragua, El Salvador, Honduras, DR, Peru, Chile^a^	N/A
Google Scholar, PubMed, Web of Science, Scopus	Smith et al (2009)	*N*=1	NR	Female	Latino values regarding end-of-life care and bereavement influences quality of care.	Central America	NR
Cambridge Core, Google Scholar, PubMed, Scopus, CINAHL	Nuñez et al (2019)	*N*=29	*M*=47.5, SD=14	48.3% female	Hospice staff need culturally competent training on importance of cultural values (simpatía, familismo) in Latino communities	NR	NR
Google Scholar, PsycNet	Grabowski and Frantz (1993)	*N*=100	*M*=47, SD=NR	69% female	Latinos who experienced an unexpected death had higher grief intensity than other groups.	Puerto Rico, Guatemala, Colombia, DR, El Salvador, Mexico, 6% NR	NR
Google Scholar	Diaz-Cabello (2004)	NR	NR	NR	Group prayer and religious rituals as essential part of grieving.	NR	NR
Google Scholar	Cann (2016)	NR	NR	NR	Cultural values and rituals allow for continuing bonds	NR	NR
Google Scholar	Lipscomb and Salinas (2020)	*N*=10	*M*=43, SD=NR	70% female	Lack of cultural support and distance from family is a risk factor for poor bereavement adjustment.	Undocumented/Temporary Protected Status; subgroup NR^*^	NR
Google Scholar	Rosa and Fuentes (2020)	NR	NR	NR	Acculturation impacts Latino/a caregiving roles.	NR	NR
EBSCOHost, Google Scholar	Schoulte (2011)	NR	NR	NR	Strategies for mental health providers to better support Latino/a mourners.	NR	NR
PubMed, Scopus, CINAHL, Google Scholar	Garcini et al (2021)	*N*=19 studies	NR	*n*=6 studies 100% female; *n*=5: 69–84% female; *n*=8 NR	More rigorous research needed to better understand bereavement adjustment for minority groups.	Majority Mexican American, NR	NR
Google Scholar, EBSCOHost, PubMed, Scopus	Doran and Hansen (2006)	*N*=9	NR	NR	Eight ways of maintaining continuing bonds: Dreams; Storytelling; Keepsakes; Sense of presence; Faith-based connections; Proximity connections; Ongoing rituals; Pictorial remembrances	Mexican American	NR
Google Scholar	Sanchez (2009)^66^	*N*=15	*M*=66.8, SD=NR	100% male	Familial support and cultural traditions vital for bereaved spouses.	Mexico, Puerto Rico, Guatemala	NR
Google Scholar, EBSCOHost, PubMed, Scopus, CINAHL	Oljtenburns (1998)	*N*=100	*M*=19.8, SD=NR	72% female	Mexican group showed higher scores of somatization and loss of control compared with whites.	Mexican American^a^	NR
Spirituality							
Google Scholar, PubMed, CINAHL, Scopus	Campesino and Schwartz (2006)	*N*=95	*M*=46, SD=NR	Female	Strong presence of spirituality in Latino culture.	Mexico, Puerto Rico, Central/South America, Cuba, Other	N/A
Google Scholar, PubMed, EBSCOHost, Web of Science, CINAHL, Scopus	Monserud and Markides (2017)	*N*=385	*M*=72.7, SD=5.5	65% female	Depression increases before and during widowhood. More frequent church attendance was protective prewidowhood	Mexican American	N/A
Grief and Immigration							
Wiley, EBSCOHost, Google Scholar, PubMed, Scopus, CINAHL	Nesteruk (2018)	*N*=56	*M*=64, SD=NR	77% female	Key themes: caregiving in transnational families; coping with loss and transnational grief; family continuity and anticipatory grief	Immigrants from Mexico, Argentina, Peru^a^	NR
Google Scholar, EBSCOHost, Scopus, CINAHL	Bravo (2017)	*N*=12	NR	NR	Importance of funeral attendance. Separation from family in bereavement worsens emotional experience.	Undocumented immigrants: Mexico, El Salvador, Costa Rica	NR
Google Scholar, EBSCOHost, PubMed, Scopus, CINAHL	Garcini et al (2020)	*N*=248	*M*=37, SD=NR	69% female	Losing a loved one from afar associated with feelings of sadness and guilt.	Undocumented Mexican immigrants	*N*=55: ≤10 years; *N*=125: 11–20 years; *N*=66≥20 years
Google Scholar, Web of Science, Scopus	Mas-Giralt (2019)	NR	NR	NR	Transnational bereavement impacts wellbeing, guilt, and anger.	Latino American and Latino British immigrants	NR
COVID							
Google Scholar, PubMed, Scopus, CINAHL	Wallace et al (2020)	NR	NR	NR	COVID-19 pandemic and “mass disenfranchised grief” for all cultures.	NR	NR
PubMed, Google Scholar, Scopus	Núñez et al (2020)	NR	NR	NR	Strategies to address challenges for those at-risk during the pandemic	NR	NR
Postdeath Rituals							
Google Scholar, Scopus	Gamino et al (2000)	*N*=74	*M*=50.7, SD=14.6	78.4% female	Mourners reporting funeral services as comforting exhibited lower grief misery	NR	NR
Google Scholar, Wiley, PubMed	Hidalgo et al (2021)	*N*=61	*M*=35, SD=9.0	NR	Supports examples of Latino death rituals in bereaved parents.	Haiti, Cuba, Puerto Rico, DR, Bahamas, Mexico, Nicaragua, El Salvador, Honduras, Ecuador, Chile, Peru, Columbia^a^	NR

^a^ Studies, including non-Latino groups in the sample.

DR, Dominican Republic; NA, not applicable; NR^*^, not reported.

**Table 3.** Overview of Literature and Strength of the Evidence Examining Bereavement in Latino/a Persons

Topic	Authors	Study design	Mode of data collection	Data source	Checklist	Strength: qualitative	Strength: quantitative	Overall strength assessment
Grief from a Cultural Lens	Hardy-Bougere (2008)	Narrative Review	Narrative Review	NA	GRADE	+		Moderate
	Clements et al. (2003)	Narrative Review	Narrative Review	NA	GRADE	−		Low
	Rosenblatt (2017)	Narrative Review	Interviews	NA	GRADE	+		Moderate
Latino Grief Experience	Brooten et al (2016)	Qualitative	Semistructured Interviews	Primary	GRADE	++		High
	Smith et al (2009)	Qualitative	Case Study	Primary	GRADE	−		Low
	Nuñez et al (2019)	Qualitative	Interviews	Primary	GRADE	++		High
	Grabowski and Frantz (1993)	Quantitative	Interviews/Survey	Primary	OCEMB		3	Moderate
	Diaz-Cabello (2004)	Qualitative	Case Study	Primary	GRADE	−		Low
	Cann (2016)	Review	Review	NA	GRADE	−		Low
	Lipscomb and Salinas (2020)	Qualitative	Interviews	Primary	GRADE	++		High
	Rosa and Fuentes (2020)	Chapter	Chapter	NA	GRADE	+		Moderate
	Schoulte (2011)	Narrative Review	Narrative Review	NA	OCEMB		5	High
	Garcini et al (2021)	Systematic Review	Systematic Review	NA	OCEMB		3	Moderate
	Doran and Hanson (2006)	Qualitative	Case Study	Primary	GRADE	+		Moderate
	Sanchez (2009)	Qualitative	Interviews	Primary	GRADE	++		High
	Oltjenbruns (1998)	Quantitative	Survey	Primary	OCEMB		4	Low
Spirituality	Campesino and Schwartz (2006)	Quantitative	Survey	Primary	OCEMB		4	Low
	Monserud and Markides (2017)	Quantitative	Survey	Secondary	OCEMB		4	Low
Grief and Immigration	Nesteruk (2018)	Qualitative	Interviews	Primary	GRADE	++		High
	Bravo (2017)	Qualitative	Interviews	Primary	GRADE	−		Low
	Garcini et al (2020)	Quantitative	Interviews	Primary	OCEMB		4	Low
	Mas-Giralt (2019)	Qualitative	Interviews	Primary	GRADE	+		Moderate
COVID	Wallace et al (2020)	Commentary	NA	NA	OCEMB		5	Low
	Núñez et al (2020)	Commentary	NA	NA	OCEMB		5	Low
Postdeath Rituals	Gamino et al (2000)	Quantitative	Surveys	Primary	OCEMB		3	Moderate
	Hidalgo et al (2021)	Qualitative	Semistructured Interviews	Primary	GRADE	++		High

GRADE, Grading of Recommendations Assessment, Development, and Evaluation; OCEBM, Oxford Center for Evidence-Based Medicine—Evidence Quality Rating Scale.

Using the CASP Checklist and GRADE Criteria, qualitative studies were generally graded to be of low-to-moderate strength, but still yielded higher quality evidence on average within that article type compared with the quantitative articles. However, quantitative findings were still considered higher quality evidence than qualitative findings that tend to use smaller samples and are impressionistic rather than employing rigorous sampling, measurement, and hypothesis testing using statistical analysis and inference. Because a substantial portion of these studies rely on ethnographic case studies and small samples, this limited our confidence in the strength and generalizability of such findings.

Most of the quantitative literature assessed through OCEMB was categorized as weak to moderate, except for one systematic review, with a key limitation being cross-sectional study designs that were unable to draw firm conclusions about causality. Thus, we conclude that the evidence base on the Latino/a bereavement experience is overall quite weak.

Nevertheless, evaluation and synthesis of the findings across the studies reviewed revealed several prevalent themes (e.g., cultural values, mourning rituals), discussed below, which can be used to inform future research efforts to build and strengthen the knowledge base in this area.

### Literature review: the Latino/a grief experience

#### Cultural values

From our review of the literature, there are a number of salient cultural values discussed that are central to the Latino/a culture, which influence both the experience and expression of grief.^19^ These values include fatalism (e.g., belief that the future is determined by God and is not under personal control), simpatía (e.g., social interactions emphasizing affection, harmony, and courtesy in interpersonal relationships), personalismo (e.g., promotion of close interpersonal relationships), familismo (e.g., strong orientation toward family, placing family needs over one's own), and allocentrism (collectivist orientation), which are all hypothesized to impact the grieving process.^20^

In a qualitative study examining the spiritual and emotional needs of 29 Latino/a patients and families in hospice, Nuñez et al^20^ highlighted the importance of Latino/a culture influences on interpersonal relationships with health care providers and spiritual and cultural preferences for receiving support at the end of life. Specifically, the authors concluded that cordial interpersonal relationships with health care providers based on the values of simpatía, familismo, and personalismo can serve as a source of emotional support that may mitigate the detrimental effects of poor psychosocial outcomes among Latino/a communities.

However, it is important to note that the impact of culture on bereavement adjustment also lies at the intersection of acculturation, with those who are less acculturated exhibiting a stronger adherence to traditional Latino/a cultural beliefs.^21^ Thus, to enhance the ability to develop targeted interventions to support the wellbeing of bereaved Latino/a persons, a more granular understanding is needed focused on what is known regarding the influence of the Latino/a culture on grief.

#### Mourning rituals

Ethnographic work surrounding culture and grief has posited that engagement in bereavement and mourning practices may promote bereavement adjustment, whereas failing to do so can heighten risk for unresolved grief.^2^ Mourning practices can provide comfort, structure, and support for the bereaved individual when they may be feeling anxious, disoriented, vulnerable, helpless, and adrift. However, most of these studies have suffered from limited methodological rigor, relying largely on narrative reviews, qualitative interviews with small samples, case studies, and anecdotal evidence—making it difficult to determine the specific impact on bereavement adjustment.

Although there is limited quantitative evidence available on the topic of Latino/a American grief, qualitative work has identified several rituals and practices in the Latino/a culture, which vary based on country of origin. One narrative review noted that mourning in Latino/a communities typically begins with an open-casket service and recitation of the rosary,^22^ and two qualitative studies highlighted that Latino/a groups rely on group prayer to promote the collective power of health and use holy water, which serves as a reminder of the Sacrament of Baptism, to guide the deceased to heaven.^20,23^ According to this literature, images of saints and Our Lady of Guadalupe (Virgen de Guadalupe) are typically placed in hospital rooms and/or the rooms of the deceased along with candles and crucifixes.

Extended wakes include food, games, candles, flowers, and other décor/offerings brought by loved ones who extend prayers intended to decrease the amount of time the deceased spends in purgatory.^24^ Furthermore, the social components of these rituals help to create and maintain continuing bonds to the deceased within the community.

Despite the importance of fulfilling mourning practices, there is considerable within-group heterogeneity in the traditions and rituals employed by the Latino/a community. In a qualitative study of Latinos, blacks, and whites examining grief at 7 and 13 months following the loss of a child, Mexican American families expressed that a wake or “velorios” provides the opportunity for the collective sharing of grief.^3^ These individuals desire a large funeral to express love for the deceased and to display strength and resilience in the family bond following death. Along with social determinants of mourning, families also honor the deceased by wearing dark colors, completing a “novena” ritual with the rosary for 9 days after the death, and avoid listening to the radio or watching television, in addition to holding mass on the first anniversary of the death. Among persons of Puerto Rican descent, deceased children may be dressed and covered in white face paint to resemble angels.^3^

Additionally, a thematic analysis of 10 in-depth interviews concluded that Latino/a mourners maintain continuing bonds with the deceased through dreams, storytelling, faith-based connections, altars, and certain types of flowers to entice the spirit of the deceased to “return home” to heaven.^25–28^ While such work is informative in providing insight into the bereavement experience within this group, factors such as generational differences in cultural beliefs and acculturation have not been explored, significantly limiting the generalizability of the knowledge base on this topic.

Research has also suggested that Latino/a family survivors have a heightened risk of complicated bereavement adjustment. One study comparing grief responses in a sample of Mexican American versus white bereaved college students found that Mexican Americans more frequently externalized their grief responses and exhibited increased physiological reactions in response to the loss.^29^ Furthermore, review of 11 observational studies investigating funeral practices on mental health and bereavement found inconclusive evidence.^30^ A study comparing bereaved people who identified as white (*n*=50) or Latino/a (*n*=50)^31^ found that Latino/a people who experience an unexpected death reported higher grief intensity but did not identify any significant associations between funeral attendance and lower unresolved grief scores. By contrast, in a sample of 211 participants (11% Mexican), unresolved grief was more likely among individuals who did not attend the deceased's funeral.^32^

Given the small number of Latino/a persons included in the sample, these results may not be generalizable. In a U.S.-based study of 74 bereaved individuals, mourners who perceived funeral services as comforting reported less grief, isolation, anger, hostility, and guilt; whereas adverse events occurring during funeral services have been associated with higher grief and other poor outcomes^33^; however, it should be noted that the sample included only 3% of individuals who identified as Latino/a, which further limits the generalizability of these findings.

#### Spirituality

When confronted with a terminal prognosis, the belief that the future is in God's control can serve as a source of comfort for both patients and families.^34^ Religious and spiritual aspects of end-of-life care and bereavement are deeply ingrained in Hispanic culture and influence the type of care wanted and deemed acceptable.^35,36^ For example, priests are viewed as meaningful and important spiritual figures who can increase feelings of comfort and negate risk for negative psychosocial outcomes.^20^ However, Latino/a patients and family members are also more likely to report that the provision of end-of-life care by providers tend to be inconsistent with patient and family wishes and fail to incorporate sensitivity to Latino/a cultural beliefs—including involvement of the family unit throughout the end-of-life care process.^37,38^ Some work has shown that Latino/a groups may avoid acknowledgment of a terminal prognosis and perceive hospice negatively because it implies that there is no hope for recovery.^38^

One study examining caregivers' perceptions of and satisfaction with hospice services indicated that those who identified as Latino/a were more likely to report that a patient's wishes regarding the setting of the death were not met, and Latino/a caregivers were more likely to be dissatisfied with emotional support provided, expressing a desire for additional spiritual and religious support.^39^ The lack of culturally sensitive services available at the end of life may be associated with Latino/as' negative feelings regarding hospice.

Latino/a groups require culturally congruent support options consistent with their beliefs and values. Specifically, the strong influence of and value attributed to Catholicism, and Christianity more broadly, which also has a strong indigenous presence (i.e., as seen in depictions of Our Lady of Guadalupe and the Virgin Mary),^24,25^ in the Latino/a culture should be reflected in the services they receive.

One study found that more frequent church attendance was associated with decreased depressive symptoms in Mexican American widows.^40^ The incorporation of and consideration of religious and spiritual influences are critical, particularly among Latino/a groups in which Catholic traditions generally dominate the bereavement process. Despite awareness of the importance of spirituality and religion in the Latino/a bereavement experience, once again the evidence is quite limited, with much of the above-referenced research relying on case studies and qualitative study designs. Further research in this area is warranted to inform the development of culturally congruent interventions that are aligned with the needs and priorities of the Latino/a population.

#### Immigrant status

Latino/a individuals may have trouble grieving due to a lack of a support network to acknowledge and socially validate their losses.^25^ In the literature examining Latino/a bereavement, perhaps the most compelling evidence relates to the study of bereavement in the context of prior immigration experiences.

In fact, the construct of ambiguous loss has been used to conceptualize the complexities of transnational immigration and family separation.^41,42^ Specifically, being separated from one's family can lead to significant psychological distress, including feelings of worry, loneliness, loss, and disconnection, as well as feelings of anger, regret, and resentment toward their home country for the circumstances compelling migrants to leave.^42^ Our microsociological theory of adaptation to loss^43^ posits that bereavement represents a state of social deprivations and psychosocial voids created by a significant interpersonal loss. The losses experienced because of immigration also create social voids (i.e., social disconnection, changes in social identity), which can lead to displacement of one's social equilibrium.

During immigration, individuals may be exposed to multiple stressors, traumas, and losses—including loss of family support and connection, loss of language, and culture, an unwelcome reception by host culture, and persistent economic hardship.^44,45^ Fear related to discrimination or detention, even for legal residents, may exacerbate social isolation and acculturative stress, all of which can lead to poor physical and mental health outcomes. Research has identified potential mechanisms that may be protective against social isolation, suffering, and distress following separation due to immigration, including increased social and family closeness, community support, religious resources and services, and other cultural rituals.^44,46^

Loss and grief are particularly salient in the context of personal experiences with immigration, which necessitates further attention regarding how immigration-related stressors may impact bereavement. For many, immigration includes feelings of loss of one's homeland and valuable relationships, idealization, and longing—typically without the presence of a support network, as well as a sense of disbelief that one has left loved ones behind, all of which are barriers to positive adjustment.^44,46^ This is also quite similar to Prolonged Grief Disorder (PGD)^47–49^ diagnostic criteria—now an official diagnosis in the DSM—which includes feelings of yearning, longing, and disbelief about the death. In fact, bereavement exhibits many similarities to the immigration experience (i.e., physical separation from loved ones) that inherently disrupts one's social identity.

#### COVID-19 pandemic and related effects

The emergence of the COVID-19 pandemic influenced in-person family and community supports for individuals of all cultures, potentially leading to “mass disenfranchised grief” given the sheer scope of and number of people impacted by the pandemic.^50^ The restriction of memorial services and funerals because of COVID-19 highlighted already persistent issues and challenges consistently experienced by Latino/a immigrant families. An immigrant in the United States, for example, who loses a loved one in their native country may not be able to attend a funeral service, which can lead to feelings of loneliness, isolation, guilt, and regret.^3^ A qualitative study^51^ documented challenges experienced by immigrants, including being forced to grieve alone because of distance from their home country and a lack of familial support.

Furthermore, immigrants also experience anticipatory grief with respect to coping with migratory losses. However, resiliency in coping with migratory-related losses may be protective in helping immigrants cope with deaths occurring in their country of origin because before a death, many immigrants anticipate never being able to see their loved one again. This may be especially relevant to undocumented individuals who do not have a choice in returning to their home country and often report feelings of hopelessness, helplessness, disappointment, and guilt.^52^

From our review, experiences with immigration may significantly influence how individuals experience, express, and resolve grief during bereavement. More research is needed to disentangle how such expressions of grief may be influenced by acculturation, country of origin, and type of loss to advance understanding of the mechanisms by which bereavement is experienced in this population. Knowledge of such factors will help facilitate the development and implementation of supportive services targeting this underserved population.

### The COVID-19 pandemic on families of Latino/a descent

As mentioned above, COVID-19 has brought the persisting issues that exist within the Latino/a community regarding physical health, mental health, and bereavement to the forefront. Data from 2020 reveal the disproportionate impact of COVID-19 on racial/ethnic minorities. Of those infected, 33% were Latino/Hispanic despite only accounting for 18.3% of the population.^53^ Furthermore, racial/ethnic minorities are generally most impacted by health disparities and experience the heaviest disease burden (e.g., lack of health insurance)^54,55^ and higher mortality rate.^56^ Disparities are further exacerbated for under-resourced immigrants who lack health insurance, experience job and/or housing instability, unemployment, low wages, language and cultural barriers, and diverse beliefs surrounding health, illness, dying, and death.^57^

The pandemic has also disproportionately affected the mental health of racial/ethnic minority groups.^58^ According to prevalence rates estimating COVID-19's impact on mental health, 40.3% of Latino/a Americans experienced depression compared with 25.3% of whites, and a greater proportion of Latino/as reported experiencing high psychological distress (28%) compared with both blacks (26%) and non-Latino whites (22%).^59,60^ Barrier Theory states that institutional and cultural barriers prevent Latino/a individuals from accessing or utilizing services,^20^ and the inequality in access to mental health services and treatments exacerbates risk for more persistent mental health issues. In 2019, 8.9 million Latinos were reported to have a mental illness and/or substance use disorder, and 25.3% of Latinos exhibited serious suicidal thoughts, with suicidal ideation four times more common among Latino/a individuals compared with their black and white counterparts.^59,61^

Moreover, COVID-19 has shed light on the pre-existing fractures and silos in the health care system that may magnify risk for adverse outcomes. Recent work has shown that immigrants are nearly two times as likely to receive extremely burdensome, aggressive end-of-life care in the final weeks of life (e.g., ventilation, cardiopulmonary resuscitation [CPR]), which typically run counter to the values and preferences of Latinos at the end of life and can impact bereavement adjustment in surviving loved ones.^62^ Other factors undermining optimal end-of-life care include linguistic challenges, lack of health literacy and health advocacy, financial hardship, perceived distrust of the health care system and providers, stigma, and discrimination.^63^

Factors that many families rely on to ensure quality and value-consistent care, including living wills, health care proxies, presence of loved ones to advocate for care, as well as the ability to understand and navigate the highly complex and bureaucratic health care system in the United States, are largely unavailable to immigrant patients and their families.^64^ With many family members abroad, many lack a significant other who can serve as a care advocate on their behalf; in cases of low health literacy, individuals lack awareness on available resources and how to access such supports, thus putting them at exacerbated risk for poor postloss outcomes in bereavement.

## Discussion

From our review and synthesis, it is apparent that Latino/a grieving and bereavement experiences cannot be understood apart from the Latino/a culture. Health care disparities, language barriers, as well as low health literacy and socioeconomic status may heighten risk for poor mental health outcomes but are also considered significant barriers to formal service utilization by Latino/a groups. Barrier Theory, which posits that institutional and cultural obstacles exist that hinder Latino/a individuals from accessing or utilizing supportive services,^20^ may help to explain Latino/non-Latino disparities in bereavement adjustment. In addition to these obstacles, there is an absence of culturally sensitive end-of-life care services, which exacerbates risk for poor bereavement adjustment.

Crosscultural misunderstanding can contribute to inadequate treatment plans that may pathologize behaviors that are considered healthy in one's culture (e.g., communicating with the dead). Despite increased awareness of the need to examine bereavement and psychosocial functioning from a cultural lens in racial/ethnic minority groups, the knowledge base on this topic is weak.

These studies are largely impressionistic and used small samples, case studies, ethnographic work, and anecdotal reports. In its current state, there is a lack of methodologically rigorous studies documenting the influence of culture on bereavement in Latino/a groups. Our review concludes that the evidence base is weak and there is a pressing need to fill large gaps in what is known about Latino/a individuals' rates of mental illness, risks, and protective factors in the context of bereavement. Basic facts about the mental health of Latino/a bereaved persons are needed before at-risk groups can be targeted and interventions developed to reduce that risk.

## Future Research

Overall, there is a limited body of research examining grief at the intersection of race/ethnicity. There is also a need to attend to the often-salient issue of immigration status that complicates bereavement adjustment. Future research should focus on the identification of Latino/a groups' mental health needs (e.g., a psychiatric epidemiology of the prevalence of Major Depressive, Posttraumatic Stress, PGDs); their significant social, cultural, and family needs (e.g., identifying needs); how fulfillment of mourning rituals and other cultural factors influence bereavement adjustment and whether they protect against disorders such as PGD, which is associated with significant distress and disability.^47^

More specifically, mixed-methods research, including a qualitative component can allow for the exploration into the meaning of culturally based rituals, beyond their theological definitions, from the perspectives of patients and their families. Furthermore, a more granular understanding is needed on what resources are wanted, needed, and/or potentially helpful in reducing the burden of bereavement experienced by Latino/a family survivors.

Policy initiatives, such as the “No Wrong Door” effort^65^ being spearheaded by state coalitions across the country, aim to streamline access to formal service options and facilitate the integration of family caregivers into the service system. However, for such efforts to be successful at decreasing disparities in resource access, further research is needed on how to effectively remove barriers to care, communication, and accessibility for those in the Latino/a community.

To develop effective interventions geared toward racial/ethnic minorities to facilitate positive bereavement adjustment, a better understanding of the interrelationships among culture, immigration, acculturation, and bereavement is needed. Furthermore, there is a need to identify and understand sociocultural barriers and resources, both of psychological (e.g., shame, stigma, guilt, lack of wanting to disclose) and structural nature (e.g., cost, transportation, work conflict) that impede the ability for those of Latino/a descent to access supportive resources. Other barriers impeding supportive service use that should also be considered include a culturally insensitive mental health care system, lack of Spanish-speaking mental health professionals, and lack of perceived effectiveness of counseling.^26^ Furthermore, in those who experience shock and impaired decision making following a death, perceived insensitivity by health care providers can interfere with the grieving process and exacerbate risk for poor postloss outcomes.^22^

Interestingly, many Latino/a individuals refuse services even when offered support or counseling^20^ and will typically exhaust other forms of social support before seeking professional help regarding grief and loss.^26^ This suggests that other forms of resources might be more acceptable and desired by this group, but also highlights the crucial need to identify and address mechanisms that can help reduce stigma and mitigate other barriers to Latino/a bereaved individuals accessing services and getting the resources needed to support their mental health.

## Abbreviations Used

CASP: Critical Appraisal Skills Programme
GRADE: Grading of Recommendations Assessment, Development, and Evaluation
OCEBM: Oxford Center for Evidence-Based Medicine—Evidence Quality Rating Scale
PGD: Prolonged Grief Disorder
